# A network analysis of anger, shame, proposed ICD-11 post-traumatic stress disorder, and different types of childhood trauma in foster care settings in a sample of adult survivors

**DOI:** 10.1080/20008198.2017.1372543

**Published:** 2017-09-19

**Authors:** Tobias M. Glück, Matthias Knefel, Brigitte Lueger-Schuster

**Affiliations:** ^a^ Faculty of Psychology, University of Vienna, Vienna, Austria

**Keywords:** Anger, rumination, shame, childhood trauma, institutional abuse, ICD-11, PTSD, network analysis, modularity

## Abstract

**Background**: Anger and shame are aspects that are specifically associated with psychopathology and maladaptation after childhood abuse and neglect. They are known to influence symptom maintenance and exacerbation; however, their interaction is not fully understood.

**Objective**: To explore with network analysis the association and interaction of prolonged, complex interpersonal childhood abuse and neglect in institutional foster care settings [institutional abuse (IA)] with anger, shame, and the proposed 11th revision of the International Statistical Classification of Diseases and Related Health Problems (ICD-11) post-traumatic stress disorder (PTSD) symptoms in adult survivors.

**Method**: Adult survivors of IA (*N* = 220, mean age = 57.95 years) participated in the study and were interviewed using the Childhood Trauma Questionnaire, the International Trauma Questionnaire, the State–Trait Anger Expression Inventory, the Displaced Aggression Questionnaire, and shame-related items. To identify the most central aspects, we used a staged network analysis and centrality analysis approach: (1) on the scale level; (2) on the item/symptom level; and (3) with modularity analysis to find communities within the item-level network.

**Results**: Trait anger, anger rumination, emotional abuse, and PTSD re-experiencing symptoms played the most important roles on a scale level and were then further analyzed on the item/symptom level. The most central symptom on the item level was anger rumination related to meaningful past events. The modularity analysis supported discriminant validity of the included scales.

**Conclusions**: Anger is an important factor in the psychopathological processes following childhood abuse. Anger rumination is closely related to PTSD symptoms; however, anger is not a part of the proposed ICD-11 PTSD in the present study.

## Abstract (zh)

**背景：**在经受童年虐待和忽视之后，愤怒和羞愧明确地与心理病理和适应不良相关联。它们也被认为可以影响系统的维持和恶化，但其中的相互作用还没有被完全理解。

**目标：**使用网络分析探索成年幸存者在寄养机构（institutional abuse, IA）长期、复杂的人际间童年虐待和忽视与愤怒、羞愧、ICD-11 PTSD症状之间的关联和相互作用。

**方法：**IA的成年幸存者（N=220，平均年龄57.95岁）参与了本研究，并使用童年创伤问卷、国际创伤问卷、状态-特质-愤怒-表达清单、转向攻击问卷和羞愧有关题目对其进行访谈。为了识别最核心的方面，我们使用了阶段化的网络分析和中心度分析方法：(1)量表水平，(2)题目/症状水平，(3)使用模块性分析在题目水平的网络中寻找社群。

**结果：**特质愤怒、愤怒反刍、情感虐待和PTSD再体验症状在量表水平有最重要的作用，并对其进行更进一步的题目/症状水平分析。在题目水平最核心的症状是和有意义的过去事件有关的愤怒反刍。模块性分析支持所包含量表的区分效度。

**结论：**愤怒是童年虐待之后心理病理过程的一个重要因素。愤怒反刍和PTSD症状紧密关联，但在本研究中愤怒没有成为ICD-11 PTSD的一部分。

## 1. Introduction

Childhood abuse (CA) and neglect pose a great risk for various mental health and psychosocial problems in the later adult’s life (Kessler et al., 2010; Norman et al., 2012). CA includes various types of violence against children such as sexual, emotional, and physical abuse and/or emotional and physical neglect (Vachon, Krueger, Rogosch, & Cicchetti, 2015). The investigation of mental health consequences related to prolonged complex interpersonal CA in institutional settings [abbreviated here as institutional abuse (IA)] started after media reports emerged on systematic IA in secular and ecclesiastical institutions such as foster care homes and boarding schools (Carr et al., 2010; Lueger-Schuster et al., 2014). IA encompasses the prolonged experience of various types of violence across childhood and adolescence in such institutions (Lueger-Schuster et al., 2014). Apart from mental health issues, such as post-traumatic stress disorder (PTSD) and other trauma-related disorders, survivors often report problems with anger and negative emotions such as shame (Carr et al., 2010; Keupp, Straus, Mosser, Hackenschmied, & Gmür, 2015; Wolfe, Francis, & Straatman, 2006). However, aspects of anger and shame have not been further investigated in the context of IA regarding their association with each other or with psychopathological symptoms. The investigation of these associations is of importance with regard to two aspects. First, the proposed criteria for PTSD in the 11th revision of the International Statistical Classification of Diseases and Related Health Problems (ICD-11) need to be evaluated within a broad array of potentially trauma-related symptoms and PTSD-triggering events. Secondly, symptom dynamics need to be better understood to further the development of transdiagnostic trauma interventions.

**Table 1.** Scales and items assessing the symptoms of anger, shame, post-traumatic stress disorder (PTSD), and institutional abuse (IA) (*N* = 220).

Measure	Subscale	Label	Symptom/item	Mean (*SD*)
DAQ	Displaced Aggression	DP		1.92 (1.14)
	Revenge Planning	RP		2.81 (1.48)
	Angry Rumination	AR		4.18 (1.39)
		AR1	When angry, focus on thoughts and feelings for long period of time	4.04 (2.02)
		AR2	Angry about certain things in life	4.45 (2.12)
		AR3	Thinking about angering events for long time	4.75 (1.91)
		AR4	Getting ‘worked up’ thinking about upsetting things in past	3.84 (2.05)
		AR5	After argument keep on fighting in imagination	3.68 (2.09)
		AR6	Ruminating about times when angered by other	3.84 (1.99)
		AR7	Helpless thinking about times when angered by other	3.97 (1.87)
		AR8	Certain long past events still cause anger	3.90 (2.14)
		AR9	Re-enacting anger episode in mind	4.23 (2.01)
		AR10	Getting caught up in anger experience	5.03 (1.71)
STAXI	Anger–In	AI		2.44 (0.71)
	Anger–Out	AO		1.89 (0.75)
	Anger Control	CO		2.85 (0.75)
	Trait Anger	TA		2.20 (0.75)
		TA1	Quick-tempered	2.18 (1.02)
		TA2	Easily upset	2.32 (1.02)
		TA3	Hotheaded	1.99 (1.04)
		TA4	Furious when criticized in front of others	2.29 (1.12)
		TA5	Infuriated for unjust evaluation	2.72 (1.07)
		TA6	Annoyed when doing something in vain	2.19 (1.06)
		TA7	Boil inside when pressured	2.27 (1.13)
		TA8	Feel like hitting when irritated	1.85 (1.09)
		TA9	Swearing when furious	2.24 (1.04)
		TA10	Angry when corrected	1.96 (0.98)
Shame	Shame Questions	SH		2.20 (1.19)
ICD-TQ	Re-experiencing	RE		1.77 (1.31)
		RE1	Distressing dreams	1.27 (1.56)
		RE2	Intrusive recollections	1.68 (1.55)
		RE3	Psychological distress at reminder	2.36 (1.50)
	Avoidance	AV		1.75 (1.38)
		AV1	Internal avoidance	1.85 (1.53)
		AV2	External avoidance	1.65 (1.56)
	Sense of Threat	TH		2.11 (1.34)
		TH1	Hypervigilance	2.46 (1.61)
		TH2	Exaggerated startle response	1.75 (1.58)
CTQ	Physical Neglect	PN		2.68 (0.78)
	Emotional Neglect	EN		4.00 (0.83)
	Physical Abuse	PA		3.11 (1.11)
	Sexual Abuse	SA		2.14 (1.18)
	Emotional Abuse	EA		3.33 (1.04)
		EA1	Called names by family	3.30 (1.32)
		EA2	Parents wished was never born	2.27 (1.36)
		EA3	Family said hurtful things	3.76 (1.11)
		EA4	Felt hated by family	3.15 (1.37)
		EA5	Feeling emotionally abused when growing up	3.72 (1.19)

DAQ, Displaced Aggression Questionnaire; STAXI, State–Trait Anger Expression Inventory; ICD-TQ, ICD-11 Trauma Questionnaire; CTQ, Childhood Trauma Questionnaire.

The complex array of sequelae calls for statistical models that are capable of grasping this complexity. So far, the scientific study of the interactions of these constructs has been dominated by the latent variable model. This model assumes that psychological constructs, such as mental disorders, are reflective and not directly observable entities that can be measured only indirectly by the symptoms they cause (Borsboom & Cramer, 2013). Recent theoretical and empirical considerations have questioned this approach and have put forward another model, the network model, as an alternative basis for modeling psychological constructs and their interactions (Borsboom, 2017; Schmittmann et al., 2013), including PTSD (Armour, Fried, Deserno, Tsai, & Pietrzak, 2017; McNally et al., 2015). In the network model, the associations of variables can be visualized and investigated in an explorative manner on the item/symptom level (e.g. Armour et al., 2017) or on the domain level (e.g. Kossakowski et al., 2015), taking their complex interactions into account. Consequently, we applied network analytical methods to investigate the aforementioned sequelae in one model to gain insight into their interplay in adult survivors of IA.

In the following sections, we review the theoretical background of the most important connections of PTSD, anger, CA, and shame to further elucidate the rationale for our study in the more specific context of IA. First, we review the associations between anger and PTSD. Secondly, we review the bivariate associations of CA with each of PTSD, anger, and shame. In a final step, we present an integrated review on the mutual connections of the above-named aspects.

### 1.1. Anger and PTSD

Anger is a known influential factor in the expression, development, and maintenance of different psychopathologies (Novaco, 2010); however, there seems to be a specificity of anger in PTSD in comparison to other anxiety disorders, with larger effect sizes in PTSD for general anger and specifically trait anger (Olatunji, Ciesielski, & Tolin, 2010). The influence of anger on PTSD and its interactions with symptoms of PTSD are not yet fully understood (McHugh, Forbes, Bates, Hopwood, & Creamer, 2012). In a network analysis, Sullivan, Smith, Lewis, and Jones (2016) found anger to be an important and highly connected symptom in the PTSD symptom network. In meta-analytical studies, anger and aggression were strongly related to PTSD and maintenance of symptoms, with the effect of anger becoming stronger over time, adding significantly to symptom distress (Orth & Wieland, 2006). In particular, anger in crime victims directed at the perpetrator and at the self was strongly associated with PTSD symptoms (Orth & Maercker, 2009). Furthermore, PTSD’s impact on anger was mediated by rumination in crime victims (Orth, Cahill, Foa, & Maercker, 2008). With regard to classification systems, the Diagnostic and Statistical Manual of Mental Disorders, Fifth Edition (DSM-5) PTSD criteria include anger as a symptom. In contrast, the proposed ICD-11 classification for PTSD does not include anger-related problems, but symptoms of anger are located with a newly introduced disorder, the proposed ICD-11 complex post-traumatic stress disorder (CPTSD) (Maercker et al., 2013). This proposed diagnosis comprises the symptoms of PTSD and additionally symptoms of affect dysregulation, negative self-concept, and interpersonal disturbances. However, previous research showed that complexly traumatized individuals do not necessarily fulfill the criteria of CPTSD, but do meet those for PTSD (Knefel, Garvert, Cloitre, & Lueger-Schuster, 2015); yet they report problems with anger. Hence, as anger also plays an important role in psychopathology associated with PTSD and not exclusively with CPTSD, the interaction of anger and the proposed ICD-11 PTSD symptoms in the context of complex trauma awaits investigation. To our knowledge, no previous study has used instruments designed to measure the proposed ICD-11 PTSD and specific instruments to measure anger; rather, single items from various measures have been used.

### 1.2. Childhood abuse and PTSD

CA is a known stable risk factor for developing PTSD (Brewin, Andrews, & Valentine, 2000). It is estimated that about one-third of victims of childhood sexual and physical abuse and neglect meet the criteria for lifetime PTSD (Widom, 1999). With regard to consequences of non-sexual CA, compared to neglect and emotional abuse, physical abuse had significant associations with PTSD (Norman et al., 2012). In general, sexual and physical abuse are the types of abuse that are strongly associated with PTSD, although they also have associations with other mental disorders, while neglect and emotional abuse have not been primarily associated with the etiology of PTSD (Cougle, Timpano, Sachs-Ericsson, Keough, & Riccardi, 2010; Spertus, Yehuda, Wong, Halligan, & Seremetis, 2003). It seems that neglect and emotional abuse are also indirect predictors of PTSD symptoms in later adulthood as they predict higher rates of lifetime trauma exposure (Spertus et al., 2003). In particular, children and adolescents who experienced complex trauma, defined as two or more experiences of sexual, physical, and emotional abuse or neglect, showed the highest odds for having PTSD symptoms (Greeson et al., 2011). Consequently, in survivors of IA who experienced prolonged interpersonal complex trauma, there is a high prevalence of PTSD and other trauma-related disorders (Carr et al., 2010).

### 1.3. Childhood abuse and anger

Anger is a common reaction in adult survivors after the experience of CA, and difficulties in anger regulation or expression have often been related to an adverse childhood history (Gardner & Moore, 2008; Hillberg, Hamilton-Giachritsis, & Dixon, 2011). Childhood physical abuse seems to predict problems with anger in adults, and levels of anger are 27% higher after physical abuse than in non-abused individuals (Springer, Sheridan, Kuo, & Carnes, 2007). It was assumed that different types of child maltreatment have differential effects on anger and aggression: while physical abuse may result in hypervigilance and attributional biases, neglect may result in difficulties with the regulation of emotion (Lee & Hoaken, 2007). Van Vugt, Lanctôt, Paquette, Collin-Vézina, and Lemieux (2014) reported that in a sample of female adolescents in residential care, anger was among the symptoms in emerging adulthood that was strongly associated with the experience of emotional abuse and neglect. In another study that investigated differential effects of the type of CA, exposure to emotional and sexual abuse alone showed moderate effect sizes compared to physical abuse, which showed only weak associations. A strong effect was reported for a combination of any two categories, while the strongest effect, again, was reported for complex exposure including all three categories (Teicher, Samson, Polcari, & McGreenery, 2006). Exposure to at least two types of abuse was also reported by survivors of IA (Carr et al., 2010), so it may be assumed that anger plays a prominent role in symptoms in our sample.

### 1.4. Childhood abuse and shame

There is consensus that shame is a common and central reaction to sexual CA and is also strongly connected to the perceived stigma related to it (Feiring & Taska, 2005; Finkelhor & Browne, 1985). Shame was proposed to be a main factor leading to poor adjustment (Feiring, Taska, & Lewis, 1996) and it seems to be a persistent emotion related to the abuse over many years and may also contribute to the maintenance of PTSD symptoms (Feiring & Taska, 2005). However, the emotional experience of shame and its relation to negative adjustment in adulthood is related not only to sexual abuse, but also to physical (Milligan & Andrews, 2005) and emotional abuse (Stuewig & McCloskey, 2005), and neglect (Bennett, Sullivan, & Lewis, 2010). Shame is an emotion that affects the whole person, related to negative self-appraisals and devaluation in relation to perceived public exposure or disapproval, leading to the desire to hide or escape from certain experiences (Tangney, 1998). This aspect of inadequacy or negative self is also strongly related to PTSD symptoms (Foa, Ehlers, Clark, Tolin, & Orsillo, 1999). Consequently, it may be assumed that shame is an even more prominent experience in survivors of IA, who not only experienced various forms of abuse and neglect, but also reported suffering from the stigma of being a ‘foster care child’ and having been treated as a ‘second class person’ throughout childhood and even their adult lives (Bruskas, 2008; Flanagan-Howard et al., 2009).

### 1.5. Childhood abuse: anger, shame, and PTSD

Apart from the above-presented mutual connections of CA, PTSD, anger, and shame, it is also important to recognize the more complex interaction of all of the aspects and further mechanisms that may influence their interconnection. Previous studies that included shame, anger, and trauma symptoms showed that they interact with each other and also predict self-harm in adulthood and behavioral problems in childhood (Bennett, Sullivan, & Lewis, 2005; Milligan & Andrews, 2005). Another aspect along with shame that connects anger and trauma is rumination. Trait anger is highly related to ruminative tendencies (Owen, 2011) and rumination is a known predictor for PTSD symptoms (Ehring & Ehlers, 2014). Shame is linked to ruminative tendencies (Lee, Scragg, & Turner, 2001), but also to anger (Taylor, 2015). This seems to be especially the case for survivors of CA, where shame moderated the effect of CA on anger for men, but not for women (Harper & Arias, 2004). Post-traumatic shame was reported to be often coupled with anger in general (Wilson, Droždek, & Turkovic, 2006) and especially predicted post-traumatic stress symptoms in victims of crime with a history of CA (Andrews, Brewin, Rose, & Kirk, 2000).

In this study, we set out to investigate aspects of anger, shame, proposed ICD-11 PTSD, and traumatic events in a sample of IA survivors with a three-phase network analysis approach. To our knowledge, no study has been conducted that incorporated all of these constructs in one analysis. As previously described, network analysis is a method capable of doing so as it takes into account the complexity of interacting aspects. In a first step, we investigated relationships on the scale level, and aimed to identify the most centrally connecting scales in the emerging network. In this first step, we aimed to investigate the interactions of the included constructs and to find the most relevant constructs in terms of centrality. We also aimed to reduce the number of items included in the next step to circumvent power problems due to our limited sample size. In a second step, we analyzed the constructs identified in the first step on the item/symptom level, and again identified the most central items/symptoms. In a third step, we used community structure analysis to assign items/symptoms to a number of identified subgroups to gain a deeper insight into the structure of the network.

## 2. Method

### 2.1. Participants

In total, 220 people (*N* = 220) aged 29–87 years (*M *= 57.95, *SD *= 9.54; 59.8% men) were included in the analyses. The majority of the sample had a lower educational background: compulsory school or less (29.6%), apprenticeship (48.9%), vocational school without A-levels (14.2%), and A-level or higher education (7.3%). Half of the participants were divorced or single (50.3%), 43.8% were married or cohabiting, and 5.9% were widowed. The majority of the sample was not employed at the time of the interview (26.9% retired, 26.5% inability to work/early pension, 16.0% unemployed and/or social assistance, 6.4% long-term sick leave, and 3.2% imprisoned); only 21.0% were employed at the time of the interview.

### 2.2. Procedure

Participants in the present study were survivors of IA in care settings under the responsibility of the City of Vienna between the years 1946 and 1986. They were recruited for the Vienna Institutional Abuse Study, which aimed to investigate the long-term correlates of IA and current health in adult survivors. Only adult survivors who disclosed their experiences to a victims’ protection commission were invited to participate in the study, and data on 220 people were included (see Knefel, Tran, & Lueger-Schuster, 2016, for a detailed description of recruitment). The study was approved by the Institutional Review Board of the University of Vienna (ref. no. 00071), and all participants gave full written informed consent.

### 2.3. Measures

We used several self-report questionnaires to assess the data. Since we included the subscales of the questionnaires in our analysis, it was important to establish their construct validity in the present sample. We thus estimated the fit of the predefined factor structure model using confirmatory factor analysis (CFA). The items were specified to load on their respective factor, and all factors were allowed to correlate within each instrument. We used the weighted least squares with mean and variance adjusted (WLSMV) estimator for robust parameter estimation and conducted the analysis using the R package lavaan (Rosseel, 2012). To evaluate the fit of the models, we used widely established criteria and benchmarks (Hu & Bentler, 1999).

#### 2.3.1. Childhood Trauma Questionnaire (CTQ)

To assess the stressor criterion for PTSD, we used the CTQ (Bernstein et al., 2003; German version, Wingenfeld et al., 2010). The CTQ is a 28-item self-report questionnaire assessing the experienced frequency of different types of CA including sexual, physical and emotional abuse and neglect on a five-point scale (from 1 = ‘never’ to 5 = ‘very often’). We separately assessed the frequency for both events that took place during foster care and events in the families. We then computed a mean score as the cumulative child abuse index for all traumatic childhood events (intrafamilial and institutional, Cronbach’s *α* = .90). To provide a descriptive overview on the types and combinations of the experienced abusive events, we dichotomized the CTQ data and interpreted values of 2 (‘rarely’) to 5 (‘very often’) as experience of a certain event, while a value of 1 (‘never’) was interpreted as no experience of the event in question. The structural model had good fit on the data, corroborating the five-factor structure of the CTQ: *χ* ^2^(df = 265) = 491.1, *p* < .001, Tucker–Lewis index (TLI) = 0.994, comparative fit index (CFI) = 0.993, root mean square error of approximation (RMSEA) = 0.063 [0.055;0.072].

#### 2.3.2. Displaced Aggression Questionnaire (DAQ)

The DAQ was developed by combining different measures of anger, aggression, and rumination (German version, Adlberger, Streicher, & Traut-Mattausch, 2009; Denson, Pedersen, & Miller, 2006). On 31 seven-point scale items (from 1 = ‘extremely unlike me’ to 7 = ‘extremely like me’), it provides a psychometrically sound measure of three distinct dimensions that predict aggressive and anger-related behavior: displaced aggression, anger rumination, and revenge planning (total scale Cronbach’s *α* = .94, subscales *α* = .88 to *α* = .94). We used the mean scores of the dimensions for the scale-level analysis. The proposed three-factor model yielded good fit indices supporting the construct validity of the DAQ: *χ* ^2^(df = 431) = 707.0, *p* < .001, TLI = 0.991, CFI = 0.992, RMSEA = 0.056 [0.048;0.063].

#### 2.3.3. Life Event Checklist for DSM-5 (LEC-5)

The LEC-5 (German version, Ehring, Knaevelsrud, Krüger, & Schäfer, 2014; Weathers et al., 2013) is a 17-item self-report questionnaire that screens for potentially traumatic events during the lifetime and, in the case of this study, asks about trauma additional to childhood trauma in families or institutions. Participants answer with ‘yes’ or ‘no’ whether they experienced a traumatic event. We used the number of experienced types of life events for the scale-level analysis. As the sum of life events assessed with the LEC can be seen as a formative measure, we did not apply CFA here.

#### 2.3.4. International Trauma Questionnaire (ITQ)

PTSD symptoms were assessed using the ITQ (Cloitre, Roberts, Bisson, & Brewin, 2013; German version, Knefel, Lueger-Schuster, & Maercker, 2013). The ITQ measures the proposed seven symptoms of ICD-11 PTSD on a five-point scale (from 0 = ‘not at all’ to 4 = ‘extremely’). It captures these symptoms on three dimensions: re-experiencing (three items), avoidance (two items), and sense of threat (two items). It can be used to estimate a self-reported ICD-11 PTSD diagnosis using the proposed criteria (Karatzias et al., 2016). A PTSD diagnosis requires a score of ≥ 2 (‘moderately’) for at least one symptom in each of the three dimensions. The ITQ showed good psychometric properties in initial evaluations (Karatzias et al., 2016, 2017; Cronbach’s *α* = .84 in this study). We used the mean scores of the dimensions for the scale-level analysis. The fit of the proposed three-factor model was good: *χ* ^2^(df = 11) = 18.2, *p* = .079, TLI = 0.993, CFI = 0.997, RMSEA = 0.054 [<0.001;0.098].

#### 2.3.5. State-–Trait Anger Expression Inventory (STAXI)

The German adaptation of the STAXI (German version, Schwenkmezger, Hodapp, & Spielberger, 1992; Spielberger, 1999) is a reliable and widely used instrument for the assessment of state and trait anger on a four-point scale (from 1 = ‘almost never’ to 4 = ‘almost always’) with 44 items. For this study, the state anger items (*n* = 10) were not included as they are normally used in experimental designs or in situations where the situational anger needs to be measured. The four STAXI trait scales measure trait anger, anger expression–out, anger expression–in, and anger control. They have also frequently been used in the study of anger in the context of trauma (Orth & Wieland, 2006; Cronbach’s *α* in the present study for trait anger *α* = .89, anger–out *α* = .90, anger–in *α* = .84, and anger control *α* = .88). We used the mean scores of the scales for the scale-level analysis. The proposed four-factor model had adequate fit: *χ* ^2^(df = 521) = 1691.1, *p* < .001, TLI = 0.956, CFI = 0.959, RMSEA = 0.102 [0.097;0.108].

#### 2.3.6. Items on shame experience

We used two self-designed questions on current shame experience that were rated on a five-point scale (from 1 = ‘almost never’ to 5 = ‘very often’) to assess self-reported shame associated with participants’ childhood/youth and with their current life: ‘When you think of your childhood/youth, do you feel ashamed?’ ‘When you think of your current life, do you feel ashamed?’ We used the mean of these items for the scale-level analysis. As this scale comprised only two items, we did not run CFA.

### 2.4. Data analysis

We used a network analytical approach to explore the relationship between anger-related aspects, shame, PTSD symptoms, and traumatic experiences. In network models, symptoms are defined to directly influence each other and thus to generate a psychopathological network of interacting elements. The elements in a network are called nodes and the associations among them are called edges. In the network models presented here, scales and symptoms are defined as nodes, and the edges can be interpreted as partial correlations among them. The edges are undirected (they do not imply any direction of prediction) and weighted with the magnitude of the corresponding partial correlation. The nodes can be investigated for their importance within the network (Borsboom & Cramer, 2013). This network approach allows for a multivariate perspective on the complex interactions among a number of scales and symptoms. We used the R package qgraph (Epskamp, Cramer, Waldorp, Schmittmann, & Borsboom, 2012) within the statistical environment R (R Development Core Team, 2016) for network estimation, visual presentation, and centrality analysis, and the R package EGA (Golino, 2016) for community structure analysis. We also provide heat maps resulting from the correlation matrices in the supplementary material (Figure S1, including a more detailed description of a heat map).

### 2.5. Network estimation

In the first step, we used the five CTQ subscales, the total number of adulthood life events, the three proposed ICD-11 PTSD symptom dimensions, the four STAXI subscales, the three DAQ subscales, and the shame scale, resulting in a total of 17 scales as nodes in the first network. After identifying the most central constructs, in the second step, we included the five CTQ emotional abuse items, the seven proposed ICD-11 PTSD items, the 10 STAXI trait items, and the 10 DAQ anger rumination items, resulting in a total of 32 items as nodes in the second network. We used the graphical lasso (glasso), a regularization technique, to estimate the network structure. In the resulting glasso network, edges can be interpreted as partial correlations between two nodes after conditioning on all other nodes in the network (conditional independence associations), e.g. the edge between the STAXI subscale trait anger and the DAQ subscale anger rumination can be interpreted as the partial correlation between those two scales after removing the effect of all the other 15 subscales in the scale-level model. The glasso technique results in a sparse network by shrinking small associations to zero to minimize the number of spurious edges, circumventing the multiple testing problem. Importantly, using glasso always results in a sparse network, even if the real network is dense. A principal idea in psychotraumatology is that traumatic events partially cause symptoms of PTSD, which in turn can be associated with other, non-trauma symptoms and related constructs (e.g. anger). We therefore assume that the network is sparse in nature and thus using the glasso is the appropriate statistical approach.

In the visualization of the networks, positive associations are printed as green (solid) lines and negative associations as red (dashed) lines. Thicker and more saturated lines represent stronger connections; thinner and more transparent lines represent weaker connections. We omitted small edges from printing by setting a minimum value of 0.03 to be included in the figure to enhance the visual interpretability.

### 2.6. Centrality analysis

For both networks, we estimated which scales or items are most central. The most used centrality measures for weighted psychopathological networks are node strength, betweenness, and closeness (Opsahl, Agneessens, & Skvoretz, 2010). Node strength is defined as the sum of weights that are connected to the focal node. A central node has strong direct connections to neighboring nodes. Betweenness defines the sum of the shortest paths between any two nodes in the network that involve this node. The shortest path between two nodes that are not directly connected runs via other nodes. When all of the shortest paths between any two nodes in the network are calculated, some nodes will lie on these paths very often (high betweenness), while others will be on the shortest path for only a few paths or not even a single shortest path in the network (low betweenness). Thus, a node with high betweenness is central in the network for information transfer and connecting nodes. Closeness defines the average distance between a node and all other nodes in the network. A node with high closeness centrality has short paths to many other nodes and quickly reacts to changes in the network. The first step of our analysis identified scales that are important in the connection of trauma, PTSD, shame, and anger-related constructs. As betweenness is the centrality measure that yields information about the importance of a node connecting the whole network, in the second step, we reanalyzed those scales on the item level that provided high betweenness centrality in the first step. We focused on the betweenness centrality measure because betweenness is especially important in a transdiagnostic network, as certain scales may act as bridges between other scales (McNally, 2016); in the second step, we aimed to investigate how this bridging effect operates on the item level.

### 2.7. Stability and accuracy

Parameters in a network may not be estimated accurately for smaller samples. We thus used the R package bootnet; we followed the procedure described by Epskamp, Borsboom, and Fried (2017) to compute 1000 bootstrapped networks and used these to estimate confidence intervals for the edge weights and the stability of the centrality metrics. Using this approach, it is also possible to estimate significant differences between centrality metrics. Epskamp et al. (2017) suggest calculating the correlation stability coefficient (CS coefficient) to quantify the stability of centrality metrics. The CS coefficient is defined as the maximum proportion of cases that can be dropped, such that with 95% probability the correlation between the original centrality metric and the centrality of networks based on bootstrapped subsets is 0.7 or higher (Epskamp et al., 2017). These authors also suggest interpreting CS coefficients of 0.5 or larger as preferable, and CS coefficients of 0.25 or larger as the minimum requirement.

### 2.8. Community structure analysis

To identify nodes that form communities (or modules), we used modularity analysis (Newman & Girvan, 2004). A community is a group of nodes that are densely connected with each other, but sparsely connected with other nodes in the network. The idea of modularity is that we estimate the extent to which the observed structure of connected nodes is statistically unexpected. The modularity index *Q* is a measure for the modularity of a network and relates the observed network structure to a randomly connected network. A *Q* value of 0 indicates no community structure and values between 0.3 and 0.7 are a good indicator of a statistically unexpected arrangement of nodes (see Newman, 2006 for a detailed discussion of the Q index; Newman & Girvan, 2004). For weighted undirected networks, the Walktrap algorithm is adequate to find communities because directions of connections are not taken into account (Pons & Latapy, 2006); it uses random walks to find groups of densely connected nodes in the network. A random walk is defined as starting from a node and randomly choosing one of the connected nodes. If there are densely connected parts in the network (corresponding to communities), those random walks tend to stay within those parts (Pons & Latapy, 2006). Golino and Epskamp (2017) developed a method, exploratory graph analysis (EGA), that uses the Walktrap algorithm to detect communities in a glasso network and is implemented in the R package EGA (Golino, 2016). This method showed robust results (Golino & Demetriou, 2017) and also provides the function bootEGA, which can obtain stable results based on bootstrapping techniques. We used 1000 bootstraps to estimate the number of communities in our data.

## 3. Results

### 3.1. Participants

The vast majority of the sample (68.6%) had experienced all types of child abuse and neglect, 30.0% had experienced four types of child abuse and neglect, and the remaining 1.4% had experienced three types of child abuse and neglect. All participants reported experiences of emotional neglect, all but one (99.5%) reported physical neglect, and also all but one (99.5%) reported emotional abuse. Physical abuse was reported by 98.2% of the sample and sexual abuse by 70.0% of the sample. The median age when they had first experienced CA was 5 years (interquartile range = 3–7 years). The median number of adult life events was five (*M* = 5.7, *SD* = 3.1, range = 0–16). More than half of the sample (54.5%) fulfilled the proposed criteria for ICD-11 PTSD. Levels of anger for the STAXI subscales trait anger (*M *= 21.99, *SD* = 7.47; *M*_norm_ = 18.08, *SD*_norm_ = 5.34), anger–out (*M *= 15.09, *SD* = 5.97; *M*_norm_ = 13.03, *SD*_norm_ = 4.02), and anger–in (*M *= 19.50, *SD* = 5.71; *M*_norm_ = 16.00, *SD*_norm_ = 4.04) were significantly higher than population norms (all *p* < .001), whereas anger control (*M *= 22.77, *SD* = 6.01; *M*_norm_ = 22.38, *SD*_norm_ = 5.27) did not differ significantly from the population norms (*p* = .334; Schwenkmezger et al., 1992). Levels of the DAQ subscales were significantly higher for revenge planning (*M* = 2.81, *SD* = 1.48; *M*_norm_ = 2.22, *SD*_norm_ = 1.16) and anger rumination (*M *= 4.18, *SD* = 1.39; *M*_norm_ = 3.45, *SD*_norm_ = 1.45), and significantly lower for displaced aggression (*M* = 1.92, *SD *= 1.14; *M*_norm_ = 2.36, *SD*_norm_ = 1.18; all *p* < .001; based on pooled norm data for 35–83-year-old individuals; Denson et al., 2006).

### 3.2. Scale-level network

**Figure 1.**
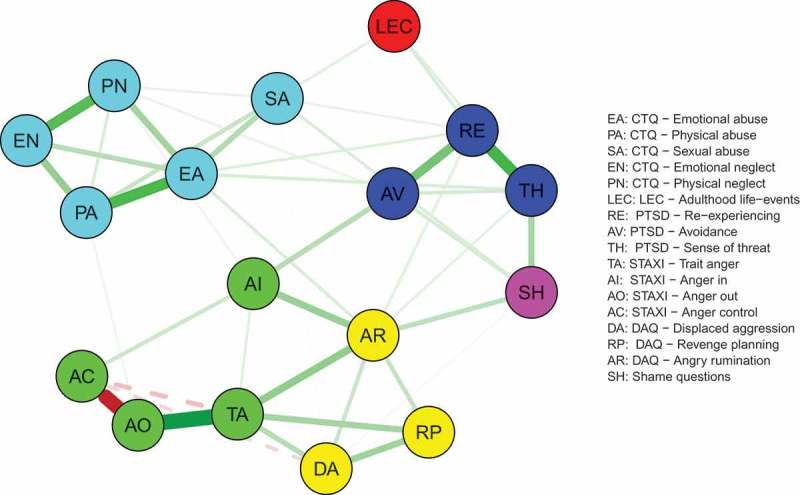
Scale-level network.

**Figure 2.**
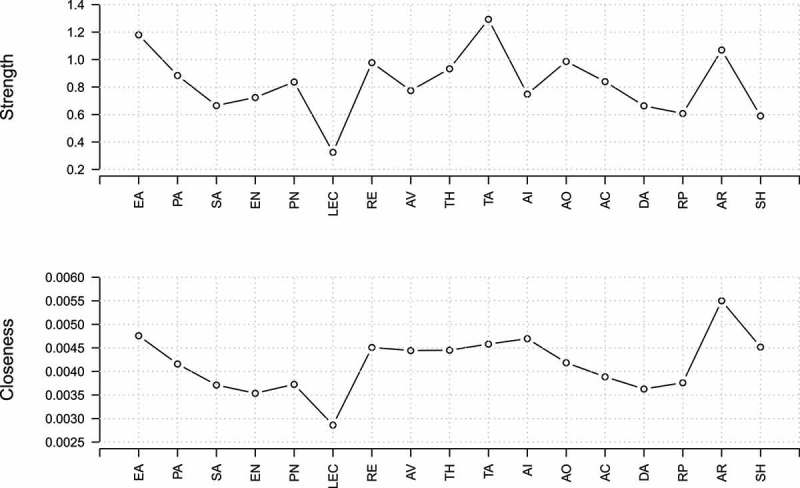
Centrality plot of the scale-level network. Strength refers to the sum of weights that are connected to the focal node; closeness refers to the average distance between a node and all other nodes in the network. For definitions of abbreviations, see Figure 1.

In a fully connected network with 17 nodes there were 136 edges. Of those possible edges, 66 were estimated to be non-zero in the glasso network (48.5%). The trauma scales were mainly connected to the PTSD scales, and to a lesser degree to the STAXI scales, the DAQ scales, and the shame scale (Figure 1). The stability analysis showed that the confidence intervals of most edge weights overlapped; however, the largest edges neither included zero nor overlapped with most other edges in the network (see supplementary material, Figure S2). The centrality analysis of the scale-level network revealed that anger rumination, emotional abuse, trait anger, and re-experiencing had the highest betweenness centrality. To test the stability of the centrality metrics, we used the CS coefficient (Epskamp et al., 2017) and interpreted values below 0.25 as unacceptable. The CS coefficient of the betweenness metric was 0.21 and the order of the betweenness metric is thus not trustworthy. We therefore also inspected the other centrality metrics (Figure 2), which were more stable: the closeness metric had a CS coefficient of 0.36 and the strength metric had a CS coefficient of 0.52. Anger rumination, emotional abuse, trait anger, and re-experiencing had relatively high values in the closeness and strength metrics. These metrics were significantly larger than the metrics of some, but not all of the other nodes in the network (see supplementary material, Figure S3). We therefore decided to include these scales as well as the remaining two scales of PTSD for the second step of the analysis on the item level. Avoidance and sense of threat were also included because our main goal was to assess the associations of PTSD symptoms with the other constructs in question.

### 3.3. Item-level network

**Figure 3.**
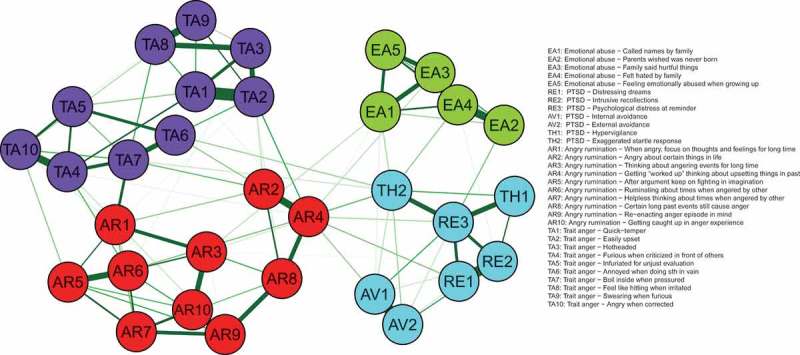
Item-level network.

**Figure 4.**
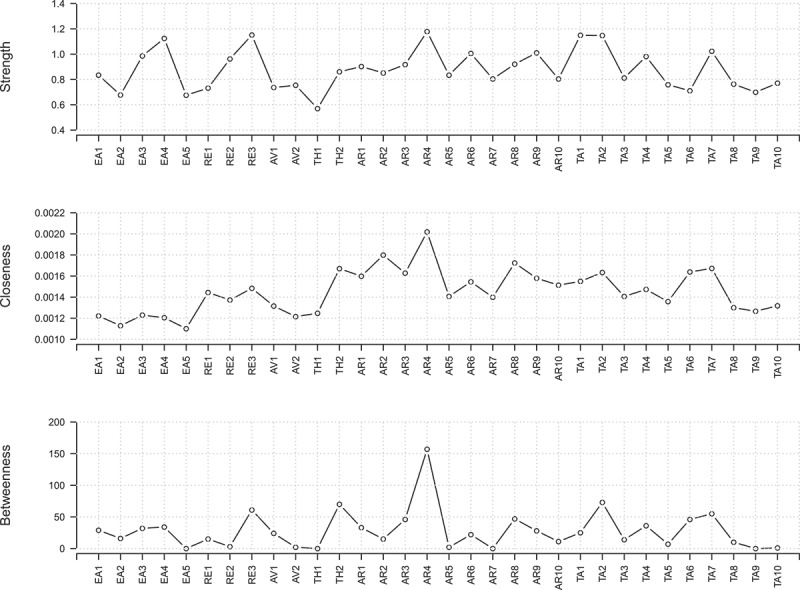
Centrality plot of the item-level network. Strength refers to the sum of weights that are connected to the focal node; betweenness refers to the sum of all shortest paths between any two nodes in the network that involve that node; closeness refers to the average distance between a node and all other nodes in the network. For definitions of abbreviations, see Figure 3.

The resulting network is visualized in Figure 3. We included all items on the anger rumination, emotional abuse, trait anger, and PTSD scales. From the 496 possible edges in this network, 165 were estimated to be non-zero (33.27%). Again, the stability analysis showed that the confidence intervals of most edge weights overlapped (Figure S4). All items were grouped closely with other items of the respective common construct. The estimated modularity index was *Q* = 0.55, indicating a community structure within the data. Six modules were found to best describe the community structure based on the results of the EGA: the first group included all emotional abuse items, the second group included all PTSD symptoms, the third group included all but three anger rumination items, the fourth and fifth groups included all trait anger items (items 1, 2, 3, 8, and 9 versus items 4, 5, 6, 7, and 10), and the sixth group was composed of the three remaining anger rumination items (items 2, 4, and 8). The stability of the centrality analysis was adequate, such that all centrality metrics can be interpreted (CS coefficients: 0.28 for betweenness, 0.28 for closeness, and 0.52 for strength). The most central node in all centrality measures was AR4 (Figure 4). Other central nodes were AR2 (closeness), RE3, TA1, TA2, EA4 (strength), and TH2 and TA2 (betweenness). However, although these nodes had significantly higher centrality metrics than some other nodes, the difference was not significant for all nodes (see supplementary material, Figure S5). All included scales and items/symptoms are displayed in Table 1.

## 4. Discussion

In this study, we combined anger, childhood trauma, proposed ICD-11 PTSD, and shame in survivors of IA in a three-phase network analysis approach. We found a network in which emotional abuse, anger rumination, trait anger, and symptoms of PTSD played a key role in the construct interaction of all scales included in the network. These aspects can be regarded as especially important for the dynamics of symptom development. On the item/symptom level, we identified that specific aspects of anger rumination seem to function as a central junction in the relationship between trauma-specific PTSD symptoms and anger. When looking at the network from a modularity perspective, we found clearly separable subgroups mostly reflecting the theoretical composition of the included constructs. Although symptoms within and between the constructs interact and presumably influence each other, they can also be clearly allocated to their scale/construct supporting their proclaimed validity. We will discuss the implications of these results in more detail in the following paragraphs dedicated to each of the separated analysis steps.

In the first step, we investigated relationships on the scale level, and aimed to identify the most centrally connecting scales in the emerging network. The most central node in terms of strength was trait anger, showing the strongest sum of connections to other nodes in the network. Trait anger was not directly connected to any type of CA or other traumatic life events. This indicated that it is not directly related to CA and traumatic life events, but may have evolved independently from external events. This is supported by research suggesting that broad biological and cognitive processes are involved in the development of trait anger (Manuck et al., 1999; Wilkowski & Robinson, 2010). However, related to the principles of the network approach, trait anger may also evolve in reciprocity with other symptoms in the network. Regardless of how trait anger develops, it appears to be a transdiagnostically important personality construct that is related to a wide range of cognitive processes maintaining or exacerbating psychopathology (Owen, 2011). As our sample consisted of adults who not only were placed in foster care as children, but also came from families with problematic backgrounds, it is likely that they had grown up in an environment with a lot of anger and aggression throughout their early lives (Greeson et al., 2011). This may be of relevance when interpreting the role of anger in the psychopathological network of our sample.

Anger rumination showed the highest closeness centrality. Closeness centrality describes the average distance between a node and all other nodes in the network, suggesting the importance of anger rumination acting as a promoter of the network. An important role of anger rumination has also been suggested by research on symptoms of borderline personality, which is also associated with CA, where it acts as a link between shame and borderline symptoms (Peters, Geiger, Smart, & Baer, 2014). This is in line with transdiagnostic research on the importance of ruminative processes for psychopathology (Aldao, Nolen-Hoeksema, & Schweizer, 2010). The clinical implication of this finding is that anger, especially connected to ruminative processes, is a central treatment target for complex trauma (Dyer et al., 2009; Ehring & Ehlers, 2014). In reducing anger rumination, owing to its centrality, it may be assumed that other symptoms connected to it will also improve when rumination is decreased (Cramer et al., 2016). The scales assessing the experience of traumatic events showed different centrality properties in the network: life events experienced in adulthood were not central, while the five types of CA had higher centrality across all centrality metrics. In particular, emotional abuse showed the highest metrics among them and also had the strongest centrality across all metrics in the network. This result supports previous research underlining the impact of emotional abuse on aggression and psychopathology (Auslander, Tlapek, Threlfall, Edmond, & Dunn, 2015; Riggs, 2010). Moreover, Spertus et al. (2003) demonstrated that emotional abuse predicted symptomatology even when controlling for other types of abuse and lifetime trauma exposure. Other research found that shame moderated adult anger and depressive symptoms after emotional abuse (Harper & Arias, 2004). Although shame had average values in some centrality metrics, the role of shame in the emerging network was below what we expected from previous research (Feiring & Taska, 2005). Shame is an important factor after trauma (Taylor, 2015); however, it is possible that experiences of shame changed over time and with regard to the disclosure and the governmental compensation process in our sample. Furthermore, shame may be a difficult construct to measure owing to various aspects that are associated with it, and because it is a challenging emotion to experience and often sought to be avoided (Gilbert & Andrews, 2010).

In the second step, we analyzed those scales on the item level that showed the highest centrality in the first step. We therefore ran a second network analysis including the items of anger rumination and trait anger, all forms of emotional abuse, and all seven proposed symptoms of ICD-11 PTSD. The modularity analysis allocated most of the included items and symptoms to their respective scales. Three items of anger rumination, ‘being angry about certain things in life’ (AR2), ‘getting “worked up” thinking about upsetting things in past’ (AR4), and ‘certain long past events still cause anger’ (AR8), formed a separate group. The items AR2 and AR4 played a key role in connecting PTSD and emotional abuse with anger. Item AR4 also had the highest index for all three centrality measures. Among all anger rumination items, those related to anger about meaningful past events and life in general (AR2, AR4, AR8) were the ones that were more closely connected to symptoms of PTSD and emotional abuse. The other items were more focused on the cognitive processes of rumination. Consequently, the trauma-related anger symptoms may also be of interest for the diagnosis of disorders specifically associated with traumatic stress. This issue is currently being discussed in relation to PTSD in DSM-5 (Durham, Byllesby, Armour, Forbes, & Elhai, 2016) and CPTSD in the proposed ICD-11 (Murphy, Elklit, Dokkedahl, & Shevlin, 2016). From a network-informed treatment perspective, it may be assumed that the anger symptoms on meaningful past events are the trauma-related promoters that activate the anger rumination subnetwork. These ‘activating’ symptoms are especially important in a symptom network (Cramer et al., 2016) and can foster a beneficial therapeutic cascade when addressed as treatment targets (McNally et al., 2015). Owens, Chard, and Ann Cox (2008) found support for the importance of considering anger in the treatment of PTSD in a veteran sample, where low levels of pretreatment anger predicted low levels of post-treatment PTSD, even for people with higher levels of pretreatment PTSD. A possible explanation could be that anger, and especially anger rumination, may be considered a maladaptive strategy to ‘erase’ or ‘undo’ trauma memories by avoiding other negative trauma-related emotions such as helplessness (Dunmore, Clark, & Ehlers, 2001), thus lowering the ability for reappraisal that in turn negatively influences PTSD symptom reduction (Kleim, Ehlers, & Glucksman, 2012). The experience of child abuse in institutional settings specifically created feelings of betrayal, powerlessness, and stigmatization (Wolfe, Jaffe, Jette, & Poisson, 2003), generating an even stronger agent for the above-proposed mechanism.

In contrast to anger rumination, trait anger was not directly connected to traumatic events (childhood and lifetime) on the scale and item levels. However, the interaction of anger rumination and trait anger should be subject of longitudinal studies, because our network was based on cross-sectional data and thus undirected. Activation may start within trait anger as a personality trait or temperament interacting with anger rumination (Wilkowski & Robinson, 2010), indirectly maintaining PTSD symptoms over activation of threat and re-experiencing symptoms (McHugh et al., 2012). This may be the underlying mechanism when looking at longitudinal studies that reported the influence of trait anger on PTSD symptoms (Meffert et al., 2008). However, it could also work in the opposite direction as other research suggests that PTSD symptoms predicted anger (Orth et al., 2008). To understand activation within a network, we consider the idea of reciprocal interaction as the best explanation of the roles of trait anger, anger rumination, and PTSD symptoms regarding maintenance and exacerbation of symptoms. Again, our sample is characterized by the experience of extensive traumatic events throughout childhood and lifetime polyvictimization, which is not the case for most other traumatized populations (Kessler et al., 2010). As the majority of our sample experienced all five types of child abuse and neglect, possible specific associations of types of abuse with adult psychopathology may be difficult to differentiate in our study.

Finally, PTSD symptoms were clustered to a single community, supporting the construct validity of the ICD-11 proposal for PTSD. Anger appears to be a related construct, but not part of the proposed ICD-11 PTSD. Emotional abuse was directly associated with several symptoms of PTSD, but only to a smaller degree with items of anger rumination and not at all with items of trait anger. This suggests a closer link between emotional abuse and PTSD than between emotional abuse and anger. This supports the convergent and discriminant validity of this revised PTSD definition that includes only core symptoms (Maercker et al., 2013).

### 4.1. Limitations

Data collection in the study was cross-sectional and conclusions on mechanisms of symptom interaction over time are hypothetical. A possible bias in our recruitment cannot be ruled out and participants in our study may differ from those survivors who did not participate. PTSD symptoms were not assessed with standardized clinical interviews. Symptoms of anger may be biased by social desirability; thus, in future research other measures that are more behaviorally oriented or based on a qualitative–narrative approach may be better suited to assess anger. It should also be considered that the instruments used may have created an artificially high within-scale covariance and an artificially low between-scale covariance based on differences between methods such as different wording or response scales. This may have biased the grouping procedure of the nodes within the networks. Furthermore, adult survivors of IA may differ from other traumatized samples, and thus generalizability of our results to victims of non-institutional CA may be limited. The reported associations in our sample may correspond more to long-term and chronic symptom patterns than to patterns found after acute or less chronic cases of child maltreatment. Finally, node centrality should be interpreted with care. It is possible that single nodes are not central in the whole network, but play an important role in their subgroup. A detailed analysis of centrality within subgroups was beyond the scope of this study and needs to be addressed in future research. Furthermore, it is also possible that single items with high centrality were missed out in our approach because we excluded the respective scale in the first step of the analysis. Thus, a larger sample with higher power would be needed for future studies.

## 5. Conclusions and implications

Emotional abuse, anger rumination, trait anger, and symptoms of PTSD interacted and influenced each other in a network on both the scale and the item/symptom levels. Trait anger and anger rumination were important factors within a network that included PTSD symptoms, but trait anger was not influenced by CA. Our findings support the idea of a narrow definition of PTSD in ICD-11. Transdiagnostic phenomena such as anger or ruminative tendencies may play a key role in symptom maintenance or exacerbation, but may not have to be included in diagnostic classifications of PTSD. As suggested from our data, there may be different ways to activate the symptom network. Future research should include longitudinal data on trait anger and ruminative tendencies before a traumatic event or, in the case of CA, either the comparison of groups or a pre–post-treatment design to identify underlying mechanisms. Our network models also demonstrate the importance of incorporating the transdiagnostic phenomena investigated here in trauma-related treatment strategies. The focus on these phenomena may even be more crucial to reducing the burden of post-traumatic mental health problems than focusing only on PTSD core symptoms.

In summary:

- We investigated the interplay of proposed ICD-11 PTSD symptoms, aspects of anger, and shame in adult survivors of childhood abuse.
- Anger is an important construct related to childhood trauma, although it was clearly distinguishable from PTSD in our study.
- Anger rumination that is related to past meaningful events is a prominent promoter in the psychopathological network following childhood trauma.

## Supplementary Material

Supplementary material
