# Supplementary material for: A network analysis of anger, shame, proposed ICD-11 post-traumatic stress disorder, and different types of childhood trauma in foster care settings in a sample of adult survivors
Source: Eur J Psychotraumatol. 2017 Sep 19;8(sup3):1372543. doi: 10.1080/20008198.2017.1372543 (PMC5632767; doi:10.1080/20008198.2017.1372543)
Supplement: Supplementary material [file ZEPT_A_1372543_SM8610.docx]

Supplementary materials

A network analysis of anger, shame, proposed ICD-11 PTSD, and different types of childhood trauma in foster care settings in a sample of adult survivors.

| 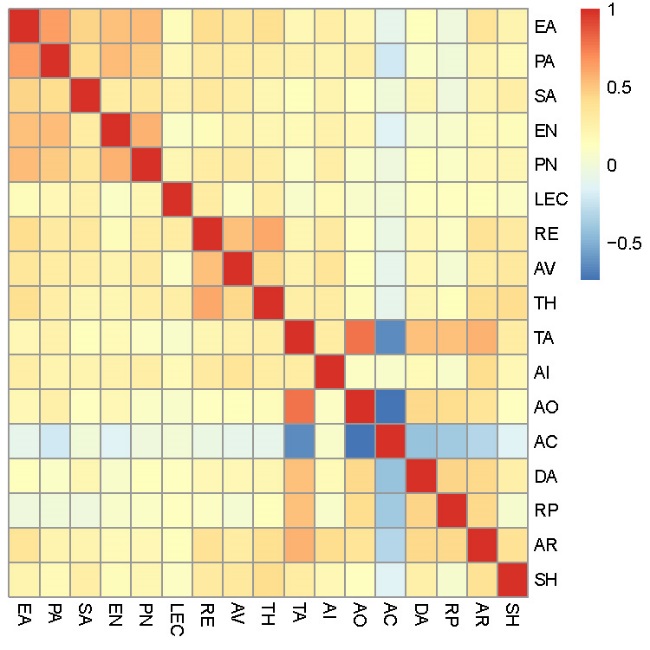  A |
| --- |
| 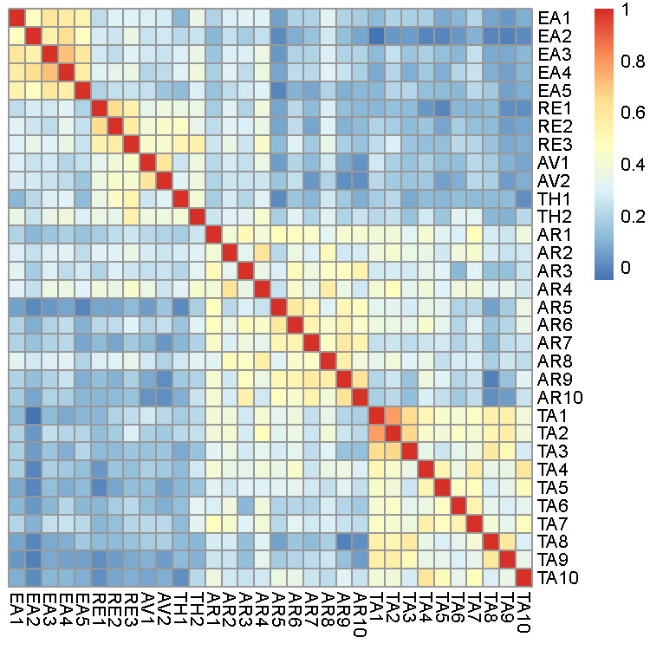  C  B |
| 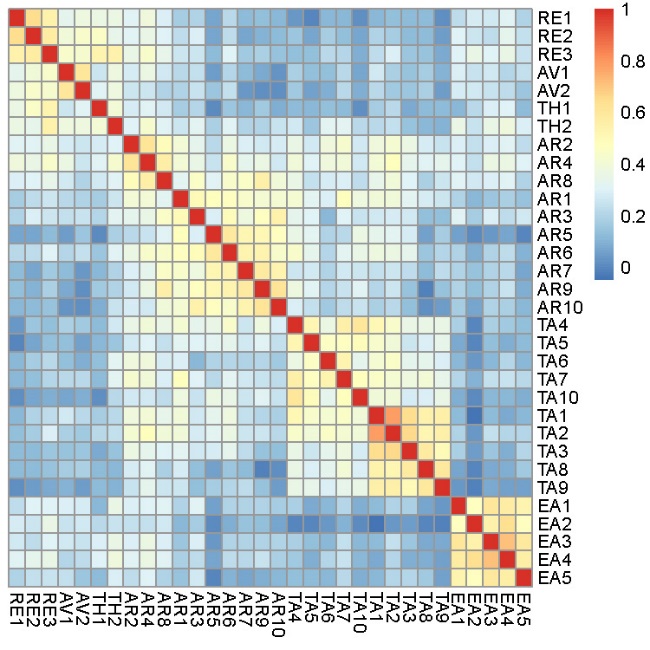 |
| Figure S1. Heatmaps of the correlation matrices. A heatmap is a graphical representation of data, where values are represented as colors. Here, warm colors (yellow to red) represent larger correlation coefficients, colder colors (white to blue) represent smaller correlation coefficients. Panel A shows the scale-level correlation matrix, panel B and C show the item-level correlation matrix, sorted according to scales (B) and to results of the community detection analysis (C). |
| 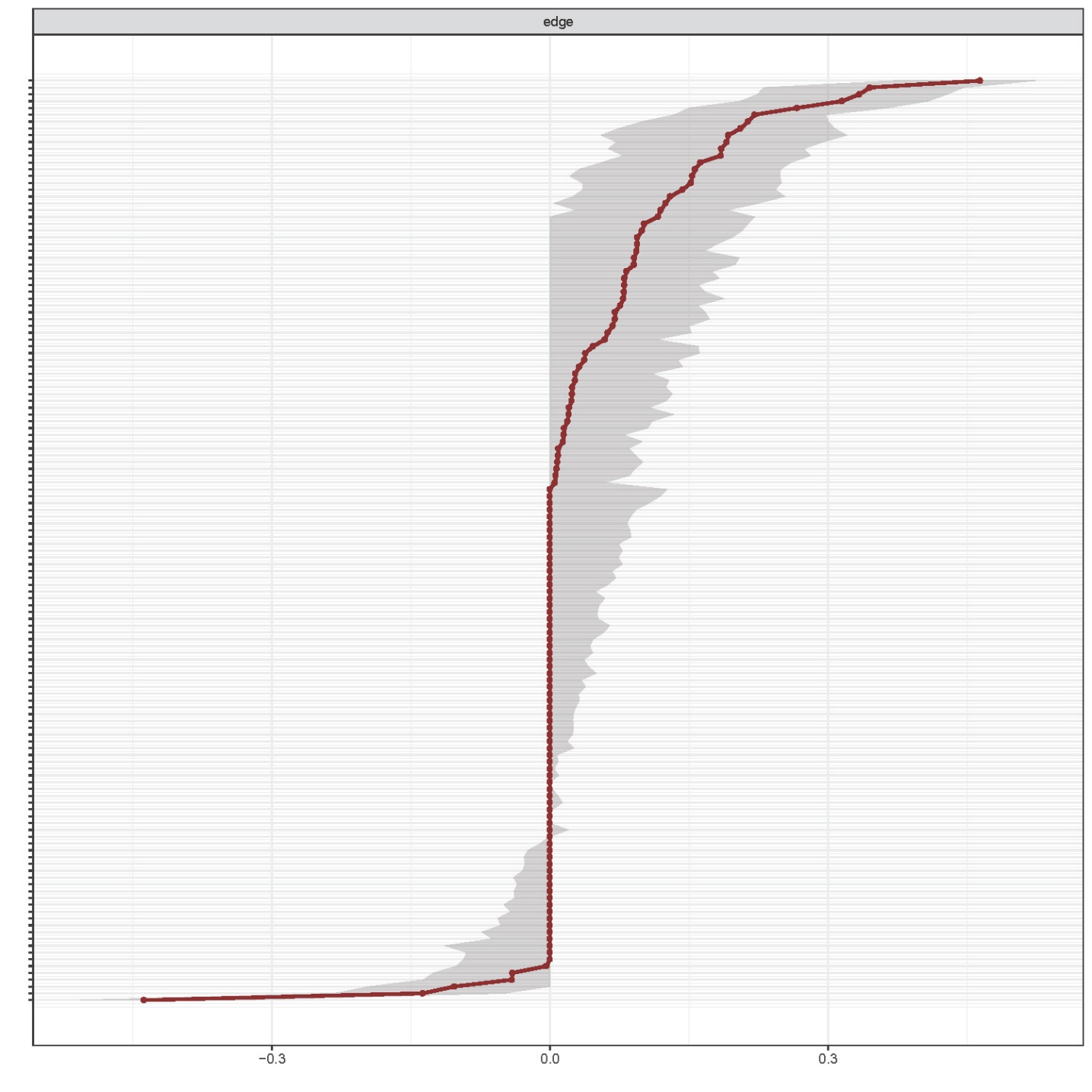 |
| Figure S2. Bootstrapped confidence intervals of edge weights of scale-level network. |

| 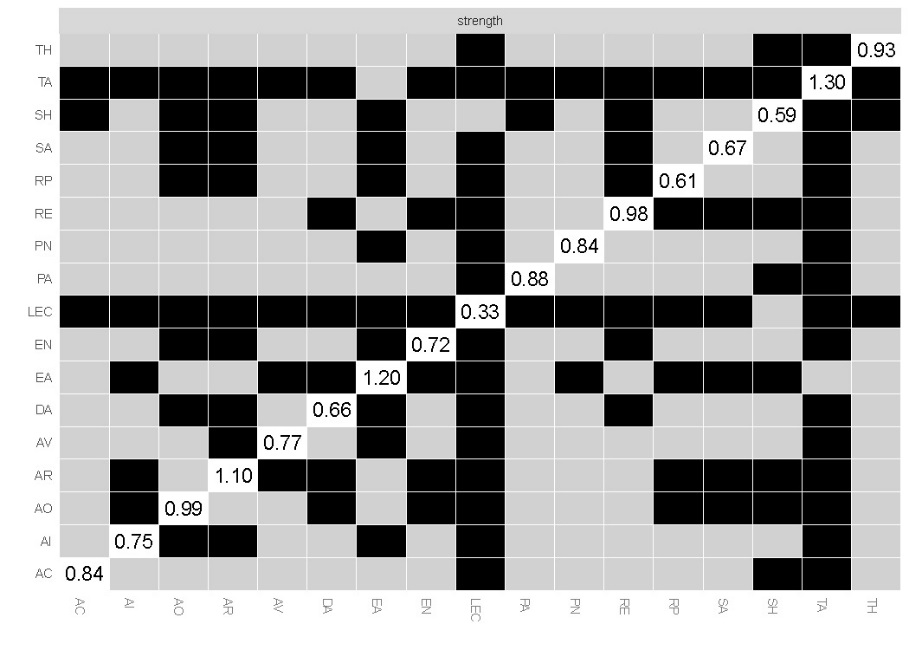  A |
| --- |
| 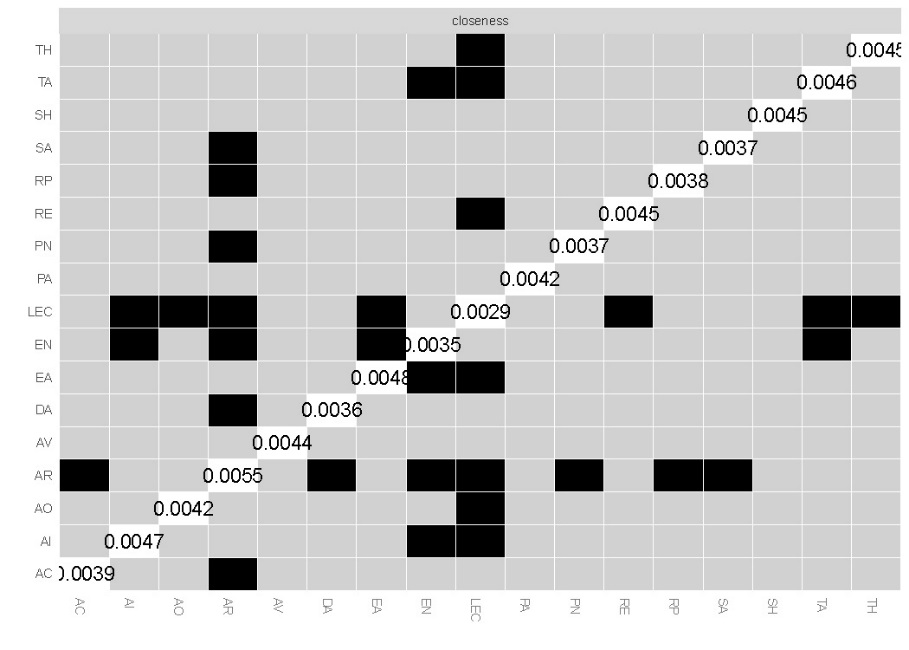  C  B |
| 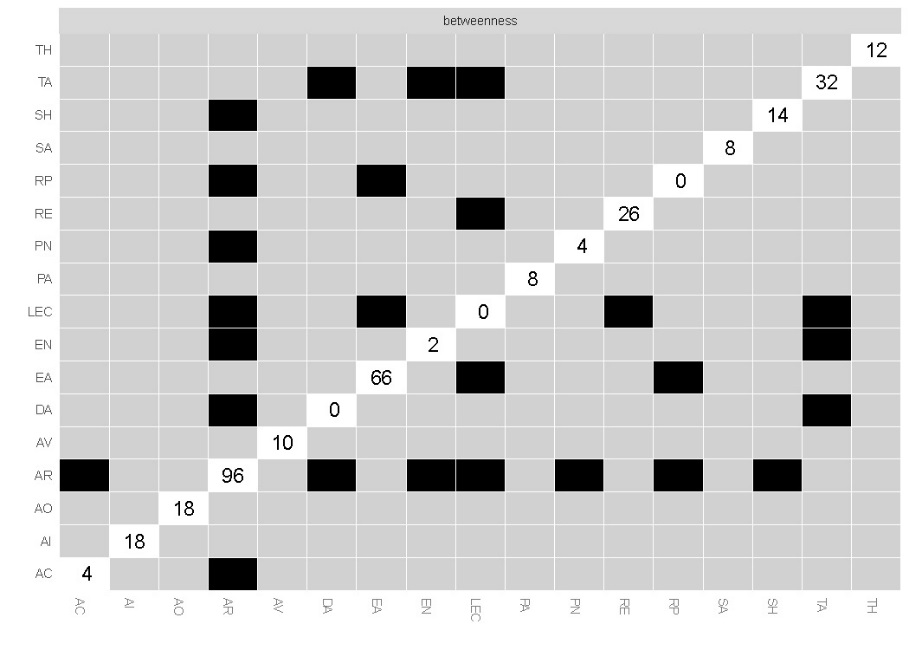 |
| Figure S3. Bootstrapped difference tests between A strength centrality, B closeness centrality, and C betweenness centrality for all nodes in the scale-level network. Values in the white boxes are centrality scores. Gray boxes are nodes that do not differ significantly from one another and black boxes represent nodes that do differ significantly from one another in the according centrality metric. |
| 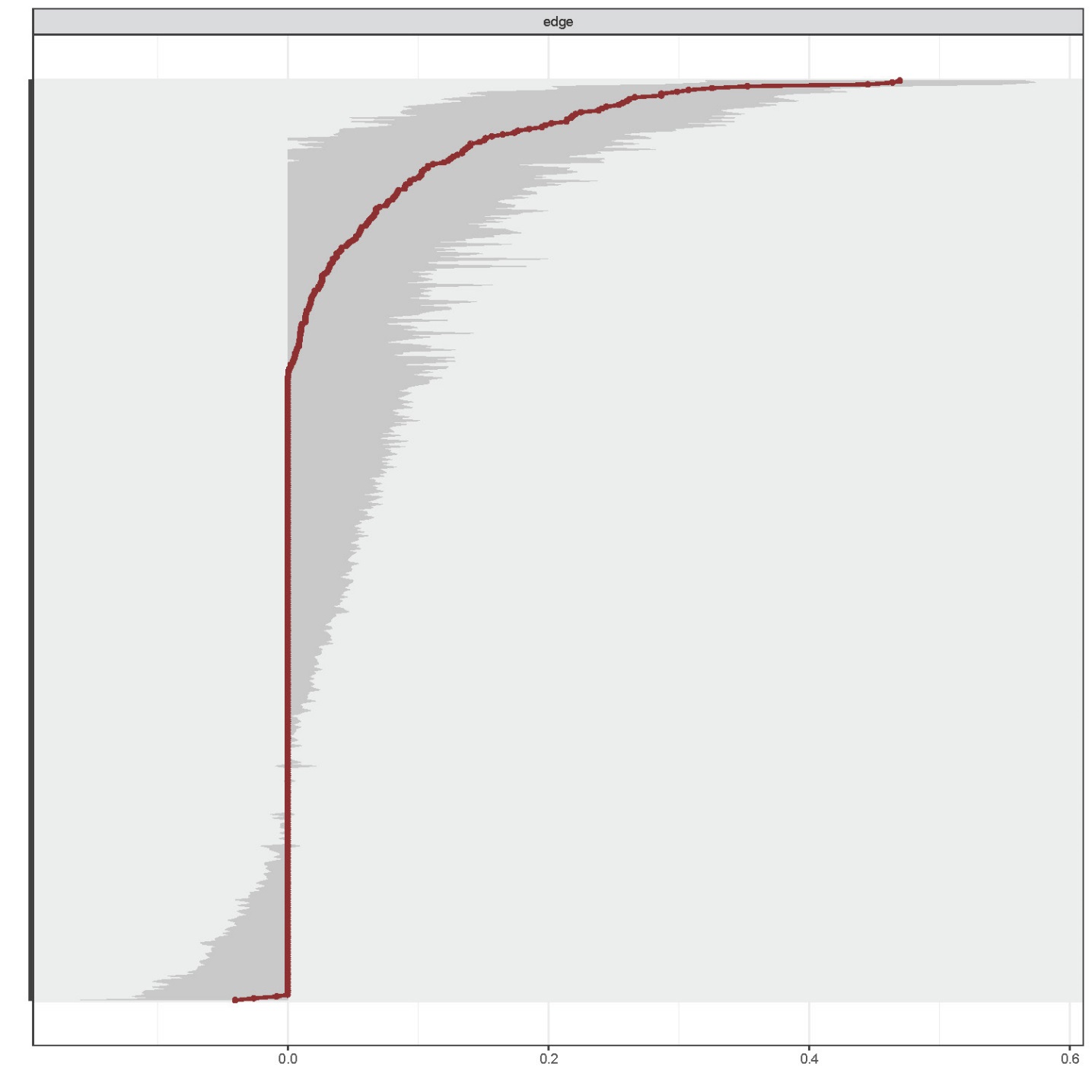 |
| Figure S4. Bootstrapped confidence intervals of edge weights of item-/symptom-level network. |

| 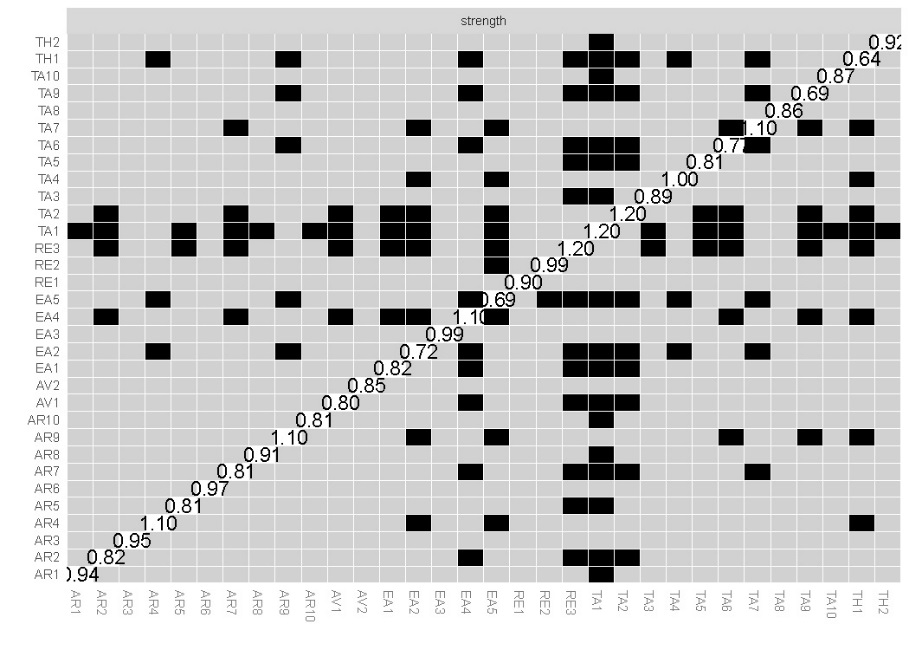  A |
| --- |
| 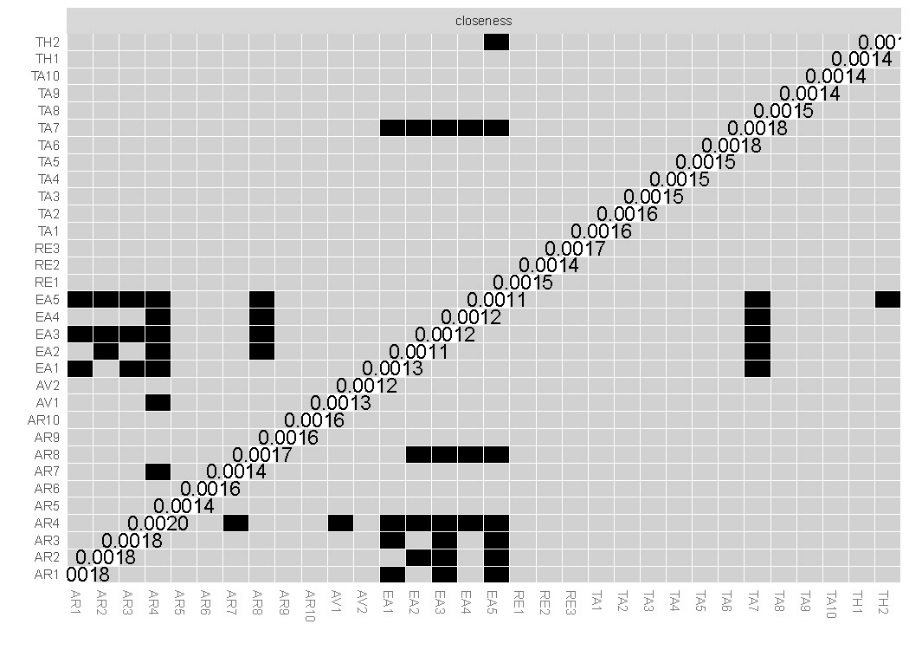  B |
| 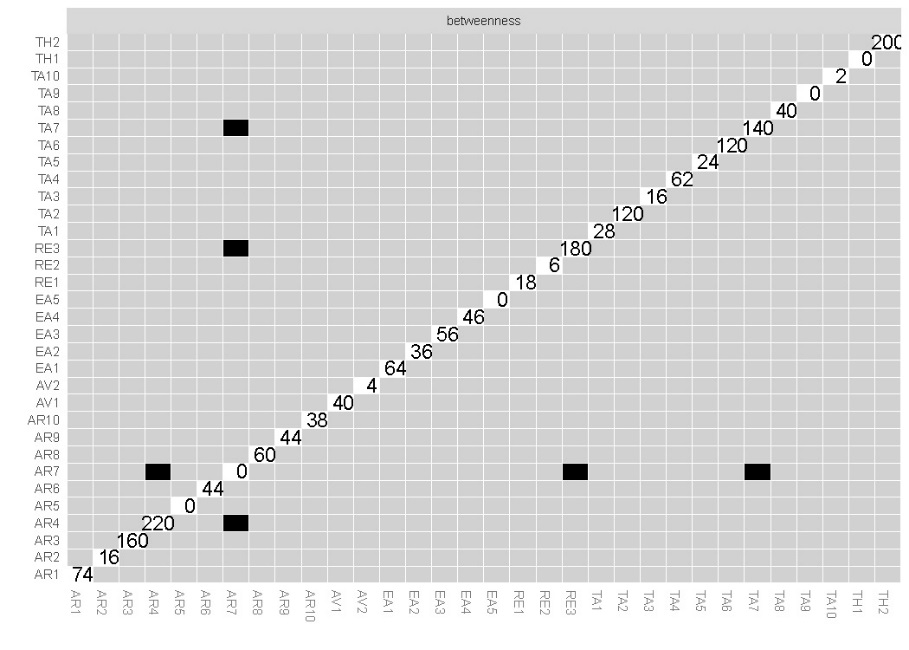  C |
| Figure S5. Bootstrapped difference tests between A strength centrality, B closeness centrality, and C betweenness centrality for all nodes in the item-/symptom-level network. Values in the white boxes are centrality scores. Gray boxes are nodes that do not differ significantly from one another and black boxes represent nodes that do differ significantly from one another in the according centrality metric. |
